# Nitric oxide of human colorectal adenocarcinoma cell lines promotes tumour cell invasion

**DOI:** 10.1038/sj.bjc.6600224

**Published:** 2002-04-22

**Authors:** A Siegert, C Rosenberg, W D Schmitt, C Denkert, S Hauptmann

**Affiliations:** Institute of Pathology, Charité Hospital, Humboldt University, Schumannstr 20/21, D-10117 Berlin, Germany

**Keywords:** nitric oxide, nitric oxide synthase II, tumour cells, monocytes, cytokines

## Abstract

The present study investigates the role of nitric oxide and the involvement of nitric oxide synthase II isoform on the invasion of human colorectal adenocarcinoma cell lines HRT-18 and HT-29. HRT-18 cells, which constitutively express nitric oxide synthase II mRNA were three-fold more invasive in a Matrigel® invasion assay than nitric oxide synthase II mRNA negative HT-29 cells. Treatment of HT-29 cells with the nitric oxide donor Deta NONOate (50 nM) as well as induction of nitric oxide synthase II mRNA and production of endogenous nitric oxide by inflammatory cytokines (IFN-γ and IL-1α) increased the invasiveness of HT-29 cells by approximately 40% and 75%, respectively. In HT-29 cells nitric oxide synthase II mRNA was also induced in co-culture with human monocytes. The invasiveness of HRT-18 cells and stimulated HT-29 cells was partly inhibited by the nitric oxide synthase II inhibitor 1400 W. These results show that nitric oxide increases the invasion of human colorectal adenocarcinoma cell lines HRT-18 and HT-29, and the involvement of nitric oxide synthase II isoform in tumour cell invasion. Therefore, the production of nitric oxide and secretion of pro-inflammatory cytokines by tumour-associated macrophages, which in turn induce nitric oxide synthase II isoform in tumour cells, promotes tumour cell invasiveness.

*British Journal of Cancer* (2002) **86**, 1310–1315. DOI: 10.1038/sj/bjc/6600224 www.bjcancer.com

© 2002 Cancer Research UK

## 

There is no doubt that tumour-associated macrophages (TAM) are an important component of the tumour stroma. They affect the behaviour of tumour cells by a variety of mediators. One of these mediators is nitric oxide (NO). NO is produced by three isoforms of the nitric oxide synthase (NOS I-III) using L-arginine as substrate. In macrophages NOS II is generally inducible by inflammatory stimuli and mediates a high-output long-lasting release of NO. Because NO is the source of reactive nitrogen intermediates (RNI), the NOS II induction is one part of macrophage cytotoxicity against tumour cells. On the other hand, NO favours neoangiogenesis, if NO concentrations do not reach a cytotoxic level (Wink *et al*, 1998). In human malignant tumours high NO concentrations have been measured *in vivo*. Although the main source of NO probably are tumour-associated macrophages, there are some reports that the synthesis of NO is inducible by cytokines in some human carcinoma cell lines like DLD-1, HT-29, A-172 and NIH:OVCAR-3 (Thomsen and Miles, 1998). The biological significance of NO in malignant tumours is not clear, but a recent study suggest that a high expression of NOS II and NOS III is associated with aggressive behaviour of colorectal adenocarcinomas (Yagihashi *et al*, 2000). The aim of the present study was to investigate if NO is able to modulate tumour cell invasiveness of human colorectal adenocarcinoma cell lines (HRT-18 and HT-29), and whether NO can be induced by cytokines produced by stromal macrophages.

## MATERIALS AND METHODS

### Monocyte isolation

Monocytes were isolated from buffy coats with Ficoll-Paque (Pharmacia, Freiburg, Germany) followed by hypotonic density gradient centrifugation in Percoll (Pharmacia) as previously described in detail (Feige *et al*, 1982). Prior co-culture experiments pooled monocytes were cultivated for 24 h in hydrophobic Teflon bags (Heraeus, Osterode, Germany) in RPMI-1640 medium (Biochrom, Berlin, Germany) with 10% human AB serum (PAA, Cölbe, Germany) and 1% glutamine (PAA) at a cell density of 2×10^6^ per ml. About 80% of these cells were monocytes, as shown by non-specific esterase activity (Sigma, Deisenhofen, Germany).

### Tumour cells

The human colorectal adenocarcinoma cell lines HRT-18 and HT-29 were obtained from the Cell Lines Service (Heidelberg, Germany) and maintained in McCOY's 5A medium (Gibco, Eggenstein, Germany) supplemented with 10% foetal calf serum (PAA).

### Co-culture of HT-29 cells and monocytes

HT-29 cells and monocytes co-culture was performed in 7.5 cm transwell plates with cell-impermeable membranes (pore size 0.4 μm, Corning Costar) in McCOY's 5A medium. Monocytes were added onto the transwell membrane above a subconfluent HT-29 monolayer in a monocyte : tumour cell ratio of 2 : 1. For RT–PCR total RNA was separately isolated from HT-29 cells and monocytes after 4, 8, 24 and 48 h of co-culture and monoculture. For nitrite determination supernatants were harvested in parallel, centrifuged and frozen at −20°C.

### Induction of NOS II in HT-29 cells

Subconfluent monolayers were cultured in serumfree McCOY's 5A medium for 24 h. Thereafter, different concentrations of human *r*IFN-γ (Sigma) and human *r*IL-1α (Tebu, Frankfurt, Germany) were added with fresh medium. After 72 h total RNA was isolated, and supernatants were collected.

### Reverse transcription – polymerase chain reaction (RT–PCR); Reverse transcription – multiplex polymerase chain reaction (RT–MPCR)

For both total RNA was isolated by the phenol/isothiocyanate method using Trizol-reagent (Gibco). RT–PCR: after cDNA synthesis efficiency was controlled by PCR for glyceraldehyde-3-phosphate-dehydrogenase (GAPDH). The primers used for amplification of GAPDH and NOS II are shown in Table 1

**Table 1:**
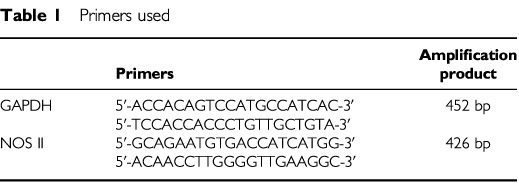
Primers used

. RT–MPCR: after cDNA synthesis, CytoXpress MPCR Kit For Human NO Metabolism Genes was used following the manufacturer's recommendations (Biosource International, Camarillo, USA) to detect mRNA of GAPDH, TNF-α, IL-1β, NOS I-III. Amplification products were separated on 2 or 3% agarose gels and stained with ethidium bromide.

### Measurement of nitrate and nitrite

Nitrate and nitrite as stable products of NO were measured with the Cayman Chemical Nitrate/ Nitrite Assay Kit (Alexis, Grünberg, Germany) in cell culture supernatants. According to the manufacturer's instruction nitrate was first converted to nitrite and than the Griess Reagents were added, which react to a purple compound. Absorbance, photometrically measured, determines nitrite concentration.

### Invasion assay

Invasion of tumour cells was determined using modified Boyden Chamber technology. Transwells with polycarbonate membranes (8 μm pore size) in six-well tissue culture plates (Corning Costar, Bodenheim, Germany) were precoated with Matrigel® Basement Membrane Matrix (Becton Dickinson, Heidelberg, Germany) diluted in serum free McCOY's 5A (1 mg ml^−1^; 675 μl per transwell) and were incubated for 2 h at 37°C. Thereafter, transwells were rinsed with serum free medium and tumour cells were seeded at a concentration of 1.3×10^5^ per ml (1.5 ml) into the transwell. NO donor or NOS inhibitor was added to the upper and to the lower compartment. After 72 h, cultures were incubated with 0.5 mg ml^−1^ 3-(4,5-dimethylthiazole-2-yl)-2.5-diphenyltetrazolium bromide (MTT) for 4 h, as described (Imamura *et al*, 1994). Invasive cells attached to the bottom of the polycarbonate membrane were scrapped off and rinsed into the lower tissue culture well with DMSO. After that, noninvasive cells in the transwell were disolved with DMSO. Formazan solutions were transfered in a 96-well microplate and absorbance was measured at 490 nm and 690 nm using an ELISA reader. For each cell line a standard curve was generated to compare invasiveness of different cell lines. Absorbance was plotted against certain numbers of cells and linear regression analysis was performed. Linearity between the absorbance and the number of cells was seen within the range of 100 to 5×10^4^ cells per well. All assays were done in triplicate.

### NO donor and NOS II inhibitor

Deta NONOate (NO donor) and 1400 W.2HCL (NOS II inhibitor) were from Alexis (Grünberg, Germany). Deta NONOate was added every 24 h and 1400 W only once at the beginning of an assay. Both chemicals showed no effects on proliferation and viability in the used concentrations as seen in direct cell counting using a CASY cell counter (Schärfe Systems, Reutlingen, Germany).

### Statistics

All data shown are from at least three independent experiments and are expressed as mean±s.d. Statistical significance was determined by using Student's *t*-test. *P*-values smaller than 0.05 were considered to be significant.

## RESULTS

### NOS isoform expression in colorectal adenocarcinoma cell lines HRT-18 and HT-29

**Figure 1:**
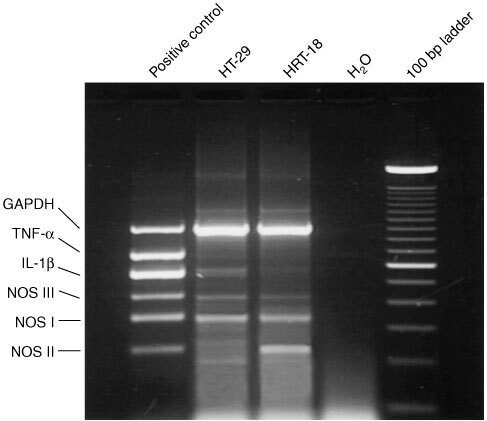
Multiplex RT–PCR analysis of NOS isoform expression. The presence of NOS I and NOS III mRNA was shown in both human colorectal adenocarcinoma cell lines HT-29 and HRT-18, NOS II mRNA was only expressed in HRT-18.

The human colorectal adenocarcinoma cell lines HRT-18 and HT-29 were used to investigate the role of NO in tumour cell invasion. The cell lines differ in the expression of the NOS II isoform. As shown by RT–MPCR both cell lines constitutively express NOS I and NOS III mRNA, but HRT-18 cells constitutively express NOS II mRNA, too (Figure 1

).

Under non-stimulatory conditions HRT-18 cells generate more NO, measured as nitrite, than HT-29 cells which can be explained by the difference in NOS II expression (Table 2

**Table 2:**
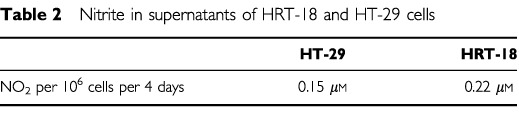
Nitrite in supernatants of HRT-18 and HT-29 cells

).

### Induction of NOS II mRNA in HT-29 cells with a combination of IFN-γ and IL-1α

**Figure 2:**
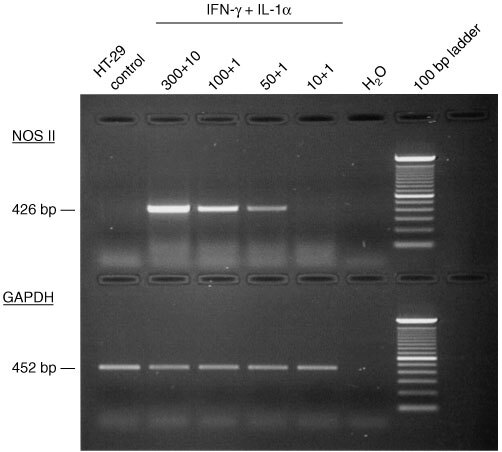
Induction of NOS II mRNA expression in HT-29 cells by cytokines. Cells were treated for 72 h with different concentrations of a combination of human *r*IFN-γ (300–10 U ml^−1^) and human *r*IL-1α (10–1 ng ml^−1^). RT–PCR showed a cytokine concentration-depending induction of NOS II mRNA.

In HT-29 induction of NOS II mRNA was achieved after incubation with a combination of IFN-γ and IL-1α in a concentration dependent manner as shown by RT–PCR (Figure 2

). A strong cytotoxic effect was observed with 300 U ml^−1^ IFN-γ and 10 ng ml^−1^ TNF-α, which correlates with a strong NOS II mRNA expression.

### Induction of NOS II mRNA in HT-29 cells after co-culture with human monocytes

**Figure 3:**
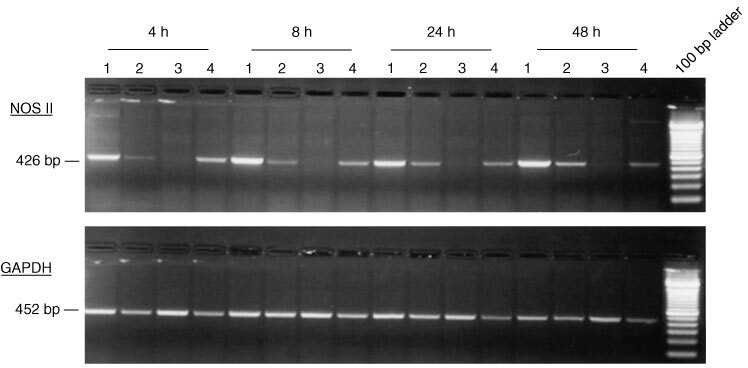
NOS II mRNA expression in HT-29/ monocyte co-cultures. RT–PCR of HT-29 cells (lane 1) and monocytes (lane 2) from transwell co-cultures and in parallel of HT-29 (lane 3) and monocytes (lane 4) from monocultures at different time points is shown. Induction of NOS II mRNA was detected in HT-29 cells at each time point of co-culture. GAPDH was used as an internal control.

To investigate whether monocytes are able to induce NO production in NOS II-negative HT-29 tumour cells, co-cultures were performed in a transwell system, which allows separate RNA analysis of the different cells. After 4, 8, 24 and 48 h of co-culture, RT–PCR showed a strong induction of NOS II mRNA in HT-29 cells, whereas the weak NOS II expression in monocytes remained unchanged (Figure 3

). In parallel cultures, total nitrite release was increased at each time point in co-cultures compared to monocytes and tumour cells alone (Table 3

**Table 3:**
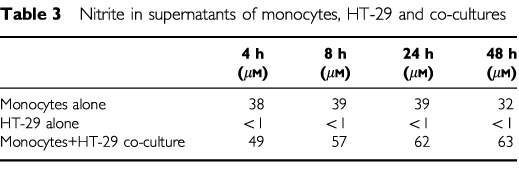
Nitrite in supernatants of monocytes, HT-29 and co-cultures

).

### Invasiveness of HRT-18 and HT-29 cells

**Figure 4:**
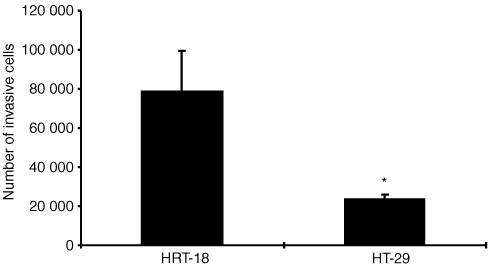
Matrigel® invasion by the human colorectal adenocarcinoma cell lines HRT-18 and HT-29. HRT-18 cells are more invasive than HT-29 cells. Cells were cultured on transwell cell culture inserts coated with Matrigel®. After 72 h cells were treated with MTT, and optical density was measured after disolving the cells with DMSO. Optical density data of invasive cells were converted into cell numbers on the basis of a standard curve for each cell line. Data of one out of three experiments are expressed as mean and standard deviation. *Significant (*P*<0.05) compared to HRT-18 cells.

Invasiveness was assessed by a Boyden chamber coated with Matrigel®. Both cell lines were invasive, but the level of invasion in the HRT-18 cell line was three times higher than in the HT-29 cell line (Figure 4

). Because a parallelism was seen between the degree of invasiveness and the amount of NO production we further investigated whether a more causative relationship exist.

### Inhibition of NO synthesis and invasion of HRT-18 cells by 1400* *W

1400 W is an irreversible inhibitor of all NOS isoforms, but has a much higher affinity to NOS II compared to NOS I and NOS III. Potency and selectivity of 1400 W to NOS II is greater than of any other described NOS II inhibitors (Garvey *et al*, 1997). 1400 W (0.5 mM) inhibited NO synthesis in HRT-18 cell line to approximately 40%, but had no effects on viability or proliferation. Cytotoxic effects were seen with 10 mM 1400 W (data not shown).

**Figure 5:**
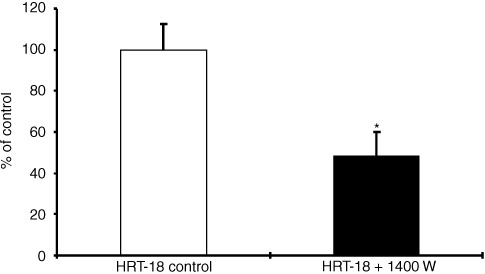
Inhibition of HRT-18 Matrigel® invasion by 1400 W. HRT-18 cells were cultured on transwell cell culture inserts coated with Matrigel® and treated with the NOS II inhibitor 1400 W (0.5 mM) for 72 h. Data of invasive cells, mean±s.d. of three independent experiments are shown. *Significant (*P*<0.05) compared to HRT-18 untreated cells.

Inhibition of NO synthesis by 1400 W was accompanied by a significant reduction of the Matrigel® invasion of the HRT-18 cell line of about 50% (Figure 5

).

### Stimulation of invasion of HT-29 cells by Deta-NONOate

**Figure 6:**
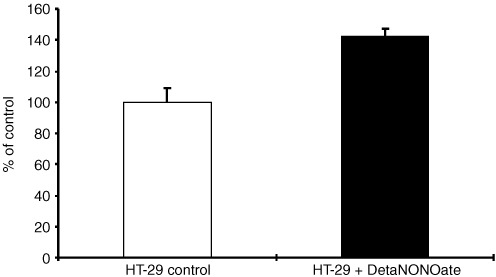
Enhancement of HT-29 Matrigel® invasion by Deta NONOate. HT-29 cells were cultured on transwell cell culture inserts coated with Matrigel® and treated with the NO donor Deta NONOate (50 nM) for 72 h. Data of invasive cells, mean±s.d. of three independent experiments are shown.

Deta NONOate is a NO donor with a half life of 20 h (Keefer *et al*, 1996). Two moles NO were released per mole of Deta NONOate. Additional exogenerously derived NO by 50 nM Deta NONOate every 24 h to NOS II negative HT-29 cells increased the number of invasive cells about 40% (Figure 6

). This concentration had no effects on viability. Cytotoxic effects were seen with 100 μM (data not shown).

### Stimulation of invasion of HT-29 cells by a combination of IFN-γ and IL-1α

**Figure 7:**
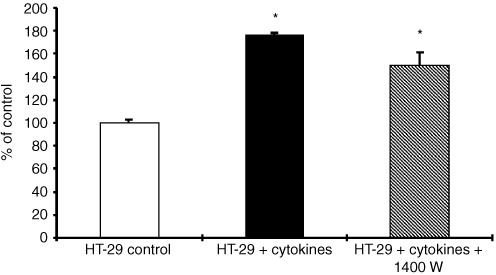
Enhancement of HT-29 Matrigel® invasion by cytokines. HT-29 cells were cultured on transwell cell culture inserts coated with Matrigel® and treated with human *r*IFN-γ (100 U ml^−1^) and human *r*IL−1α (1 ng ml^−1^) for 72 h. This enhancement was slightly abrogated by addition of the NOS II inhibitor 1400 W (0.5 mM). Data of invasive cells, mean±s.d. of three independent experiments are shown. *Significant (*P*<0.05) compared to HT-29 untreated cells.

Induction of NOS II in HT-29 cells by the cytokines IFN-γ (100 U ml^−1^) and IL-1α (1 ng ml^−1^) and production of NO by the tumour cells itself significantly increased Matrigel® invasion of HT-29 cells about 75%. This effect was marginally abolished by the NOS II inhibitor 1400 W (Figure 7

), which proves a contribution of NOS II in the process of tumour cell invasion. It also indicates, that cytokines induce NO independent mechanisms of tumour cell invasion.

## DISCUSSION

For some human cancers elevated NOS activity has been reported. The level of NOS protein and/or NOS activity has been positively correlated with the degree of malignancy in human gynaecological cancers (Thomsen *et al*, 1994), central nervous system tumours (Cobbs *et al*, 1995), breast cancer (Thomsen *et al*, 1995), squamous-cell carcinomas of the head and neck (Gallo *et al*, 1998), lung cancer (Fujimoto *et al*, 1997) and oesophageal squamous cell carcinoma (Tanaka *et al*, 1999). Obviously, NO plays an ambivalent role in tumorigenesis depending on NOS activity and resulting NO concentrations. Although there are some reports showing induction of tumour cell apoptosis and cytostasis by high levels of NO (Xie and Fidler, 1998), an increasing number of reports shows a positive correlation between NO and tumour progression (Thomsen and Miles, 1998) explained by NO-stimulated migratory, invasive and angiogenic abilities of tumour cells (Thomsen and Miles, 1998; Lala and Chakraborty, 2001).

This study clearly demonstrates, that NO increases the invasion of the human colorectal adenocarcinoma cell lines HRT-18 and HT-29. HRT-18 cells and HT-29 cells differ in NOS expression and have different invasive capacities. The NOS II negative HT-29 cells are less invasive indicating a role of NOS II in Matrigel® invasion. The invasiveness of HT-29 cells was further enhanced by the exogen addition of NO or the induction of endogen NO production by cytokines. In general, NOS II is inducible in a lot of human tumour cell lines by pro-inflammatory cytokines (Thomsen and Miles, 1998). It has been shown too, that bacterial infection of human colon epithelial cells is a sufficient stimulus to upregulate their expression of NOS II and NO production (Witthoft *et al*, 1998). Enhanced expression of NOS II and NOS III has been recently discussed to correlate with tumour growth and vascular invasion in human colorectal cancer (Yagihashi *et al*, 2000; Lagares-Garcia *et al*, 2001). In our study the involvement of the NOS II isoform in Matrigel® invasion of the colorectal adenocarcinoma cell lines was evidenced by the inhibition of invasion by 1400 W, the most selective NOS II inhibitor reported to date. 1400 W never completely abolished the basal invasion of HRT-18 or the cytokine-stimulated invasion of HT-29, which indicates the existence of other invasion-inducing mechanisms beside the NOS II/NO pathway.

Not investigated in this study were mediators of the NO-stimulated invasion, which lead to essential steps as migration and matrix degradation. Reduced migratory and invasive capacities of the murine mammary cell lines C3L5 and C10 were demonstrated with the NOS inhibitor L-NAME (Jadeski *et al*, 2000). Furthermore, NO promotes tumour cell invasion in the C3L5 murine mammary adenocarcinoma model by altering the balance between the expression of matrix metalloproteinase-2 (MMP-2) and it's tissue inhibitors-2 and -3 (TIMP 2 and TIMP 3) (Orucevic *et al*, 1999). Activation of MMP's by NO has also been shown in chondrocytes (Murrell *et al*, 1995). Together, MMP's and TIMP's modulate the structure of the extracellular matrix (ECM) and changes of their expression level or activation of MMP's weaken the integrity of the ECM, which is a prerequisite for invasion and metastasis (Kohn and Liotta, 1995).

Tumour-associated macrophages are the source of various cytokines within malignant tumours, which can induce NOS II mRNA expression. There is an earlier report describing the induction of NOS II mRNA in co-cultures of the human urothelial bladder carcinoma cell line RT4 and human monocytes (Konur *et al*, 1996). In co-cultures of the human colorectal adenocarcinoma cell line HT-29 with human monocytes we found a similar strong induction of NOS II mRNA in the HT-29 cell line, but no change in the basal NOS II mRNA level in the monocytes. Therefore, the enhanced nitrite concentration in the co-culture supernatants probably result from NO synthesis in HT-29 cells, which has no obvious cytotoxic effect until 48 h of co-culture. It is known from rodents, that NO-derived reactive nitrogen intermediates (RNI) produced by macrophages are cytotoxic against tumour cells (Bogdan and Nathan, 1993). But the reported NOS activity in human tumour cell lines or tumour tissues is at least 1–2 orders of magnitude lower than the enzyme activity associated with the cytotoxicity of rodent macrophages (Keller *et al*, 1990). The production of RNI by monocytes in the used co-culture model can not be proposed, since no relevant levels of RNI are produced before maturation of monocytes to macrophages (Martin and Edwards, 1993).

Tumour-associated macrophages are promoters of tumour growth and invasion by different ways. This study shows that pro-inflammatory cytokines induce NOS II expression and NO synthesis in tumour cells. NO promotes tumour progression by mechanisms like stimulation of angiogenesis (Jadeski and Lala, 1999), and as demonstrated in this study also by stimulation of invasiveness.
